# Carbon Dots Anchoring Single-Atom Pt on C_3_N_4_ Boosting Photocatalytic Hydrogen Evolution

**DOI:** 10.3390/molecules29081890

**Published:** 2024-04-21

**Authors:** Jing Wang, Jiayu Song, Xin Kang, Dongxu Wang, Chungui Tian, Qin Zhang, Hui Zhao, Jiancong Liu

**Affiliations:** Key Laboratory of Functional Inorganic Material Chemistry, Ministry of Education, Heilongjiang University, Harbin 150080, China; wj_0475@163.com (J.W.); 13349376578@139.com (J.S.); kangxin940821@sina.com (X.K.); wangdongxu@hlju.edu.cn (D.W.); tianchungui@hlju.edu.cn (C.T.); zhangaqin@163.com (Q.Z.); zhaohui202402@163.com (H.Z.)

**Keywords:** carbon dots, carbon nitride, single-atom Pt, photocatalytic hydrogen revolution

## Abstract

Carbon nitride (C_3_N_4_) has gained considerable attention and has been regarded as an ideal candidate for photocatalytic hydrogen evolution. However, its photocatalytic efficiency is still unsatisfactory due to the rapid recombination rate of photo-generated carriers and restricted surface area with few active sites. Herein, we successfully synthesized a single-atom Pt cocatalyst-loaded photocatalyst by utilizing the anchoring effect of carbon dots (CDs) on C_3_N_4_. The introduction of CDs onto the porous C_3_N_4_ matrix can greatly enhance the specific surface area of C_3_N_4_ to provide more surface-active sites, increase light absorption capabilities, as well as improve the charge separation efficiency. Notably, the functional groups of CDs can efficiently anchor the single-atom Pt, thus improving the atomic utilization efficiency of Pt cocatalysts. A strong interaction is formed via the connection of Pt-N bonds, which enhances the efficiency of photogenerated electron separation. This unique structure remarkably improves its H_2_ evolution performance under visible light irradiation with a rate of 15.09 mmol h^−1^ g^−1^. This work provides a new approach to constructing efficient photocatalysts by using CDs for sustainable hydrogen generation, offering a practical approach to utilizing solar energy for clean fuel production.

## 1. Introduction

Hydrogen evolution via photocatalytic water splitting is acclaimed as a pivotal technology for transforming solar energy into hydrogen fuel, which shows great potential in solving energy and environmental problems [1,2]. Designing efficient and economical photocatalysts is key to achieving effective solar-driven hydrogen evolution. Carbon nitride (C_3_N_4_) has been widely studied as an ideal photocatalyst due to its non-toxicity, excellent stability, low cost, and appropriate optical band gap [3,4,5]. However, its potential is restricted by limitations such as inadequate visible light absorption, a limited surface area with too few active sites, and a rapid recombination rate of photo-generated carriers [6,7]. Many efforts have been made to overcome these challenges. To tune the band gap and visible light absorption efficiency, methods such as element doping [8], defect manufacturing [9], and heterojunction construction [10] have been found to be effective. Among them, designing a special local electronic structure is key to improving its photocatalytic performance. To provide more surface-active sites for the reaction, controlling the morphologies of C_3_N_4_ for a large specific surface area is crucial [11]. Notably, a facile in situ exfoliation and conversion strategy has been developed, which can prepare layered C_3_N_4_ with a large specific surface area by using alcohol molecular insertion, thermally induced exfoliation, and condensation accompanied by constructing heterojunctions [12]. To suppress the recombination of photo-induced carriers, loading cocatalysts, such as Pt, to trap photogenerated electrons on the conduction band can accurately separate the electrons and holes and improve the photocatalytic hydrogen evolution efficiency. However, traditional methods of cocatalyst deposition often result in the formation of large Pt nanoparticles, which are less efficient in terms of atom utilization [13]. Constructing appropriate intermediaries between the metal co-catalysts and C_3_N_4_ can not only control the size of cocatalysts but also swiftly evacuate photo-induced carriers into the active sites to facilitate the photocatalytic conversion process [14,15]. Despite these advances, devising a comprehensive strategy that comprehensively boosts the performance of photocatalytic materials remains challenging. Finding a suitable mediator material to simultaneously regulate the light absorption efficiency, specific surface area, and cocatalyst loading is important for constructing economical and efficient photocatalysts.

Carbon dots (CDs) have emerged as a new class of carbon nanomaterials, drawing considerable interest thanks to their non-toxicity, adjustable light absorption spectrum, and modifiable surface structure [16]. Recently, CDs have been widely used for total water splitting, hydrogen evolution reactions, carbon dioxide reduction, and organic photocatalytic reactions [17,18,19]. When combined with C_3_N_4_, CDs can not only broaden the range of light absorption but also tune the electronic landscape of the host material [20]. The conjugated π structure of CDs acts as a platform for effective charge transfer under solar illumination, and their electron-accepting capabilities after photoexcitation significantly boost the kinetics of photocatalytic reactions [21,22]. Importantly, the diverse and modifiable surface functional groups of CDs offer unique interaction sites for metal cocatalysts [23]. Via this synergy, the localization of Pt cocatalysts at the nanoscale becomes feasible, leading to the construction of small clusters and single atoms. These finely dispersed catalytic sites exhibit excellent activity owing to their high atomic utilization efficiency and distinctive electronic properties, which optimize the catalytic processes for hydrogen production. Therefore, the combination of CDs and C_3_N_4_ shows great potential for sustainable energy production [24]. However, current studies mainly focus on exploring various heteroatom dopants to tune the band gap and constructing heterojunction structures; there is still a lack of examples to fully utilize the surface functional groups of CDs to confine the growth of single-atom cocatalysts and form sufficient contact and effective electron transfer.

Here, we synthesize a unique photocatalyst with the incorporation of CDs into the porous C_3_N_4_ matrix. The obtained CDs/C_3_N_4_ composite not only enhances the spectral absorption but also promotes the separation of photogenerated charge carriers, which greatly improves the overall efficiency of the photocatalytic reaction. Facilitated by the anchoring effect of CDs, the cocatalyst Pt presents single-atom distribution with Pt-N connection, which improves the atomic utilization efficiency of Pt and promotes electron transfer, leading to a substantial increase in hydrogen production. The Pt-CDs/C_3_N_4_ photocatalyst demonstrates a hydrogen evolution rate of 15.09 mmol h^−1^ g^−1^ under visible light, marking a 4.6-fold increase over the traditional Pt-C_3_N_4_. This significant enhancement in performance highlights its potential as a promising material for hydrogen generation.

## 2. Results and Discussion

### 2.1. Material Preparation and Characterizations

**Figure 1:**
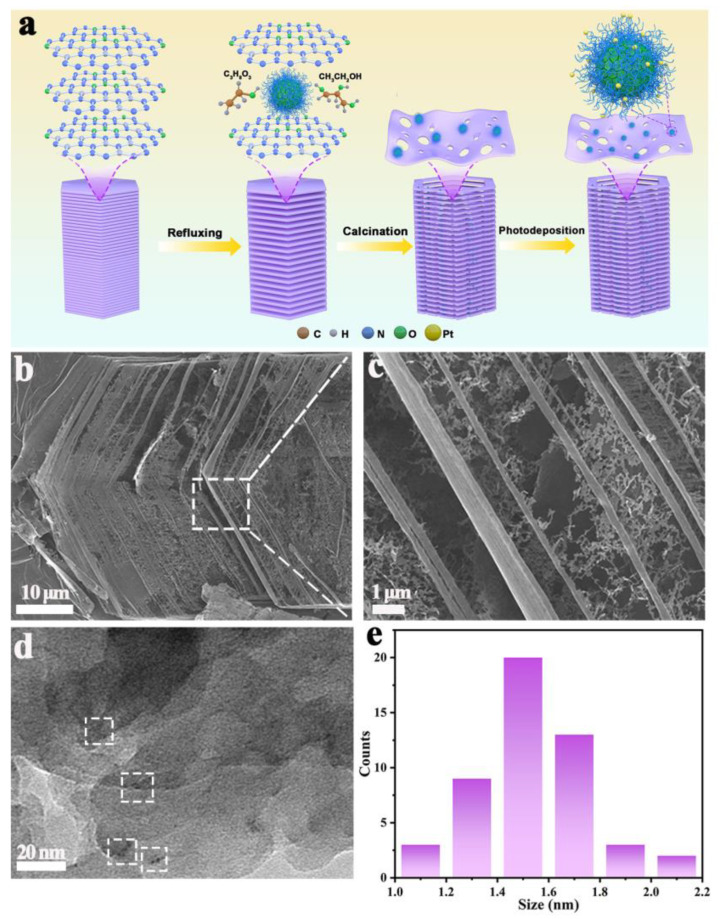
(**a**) Schematic illustration of the preparation process of Pt-CDs/C_3_N_4_, (**b**,**c**) SEM images of CDs/C_3_N_4_, (**d**) HR-TEM images of CDs/C_3_N_4_ (The white boxes highlight the loaded CDs), and (**e**) the particle size distribution map of CD nanoparticles on C_3_N_4_.

The hollow filamentary C_3_N_4_ composite loaded with CDs (named CDs/C_3_N_4_) was synthesized via a solvent-assisted reflux stripping strategy combined with CD loading (Figure 1a). Initially, supramolecular precursors of C_3_N_4_ were generated using a straightforward hydrothermal method. Subsequently, these precursors were intercalated with pre-synthesized CDs via co-refluxing with ethanol and glycerol [12]. This approach not only facilitates the entry of CDs into the molecular layers but also promotes the formation of C_3_N_4_ with a porous structure. The process concluded with a stripping and extraction step via calcination, which expanded the layer spacing of the precursors. Scanning electron microscopy (SEM) imaging of the resulting CDs/C_3_N_4_ composite exhibits a predominantly hollow, porous filament-like structure (Figure 1b,c), which is advantageous for providing more active sites and shortening the transmission path of electrons and holes within the material, thereby enhancing carrier separation [12,25]. The refluxing process involving ethanol, glycerol, and CDs leads to larger distances between layers in the precursors. This effect, combined with the interaction between the functional groups of CDs and C_3_N_4_, is responsible for the unique porous morphology. With different amounts of CD loading, the resultant CDs/C_3_N_4_ composites show similar hollow filamentary morphology (Figures S1 and S2). In contrast, the traditional bulk C_3_N_4_ (named B-C_3_N_4_) was synthesized using a widely employed polycondensation method with melamine as the precursor (Figure S3). By directly annealing the supramolecular precursors without the solvent-assisted refluxing process, C_3_N_4_ with tubular morphology (named C_3_N_4_) was synthesized (Figure S4). The high-resolution transmission electron microscopy (HR-TEM) of CDs/C_3_N_4_ further reveals the hollow filamentary structure (Figure 1d). Notably, it demonstrates that CDs with particle sizes of 1.4–1.7 nm are statistically uniformly distributed on the C_3_N_4_ (Figure 1e).

**Figure 2:**
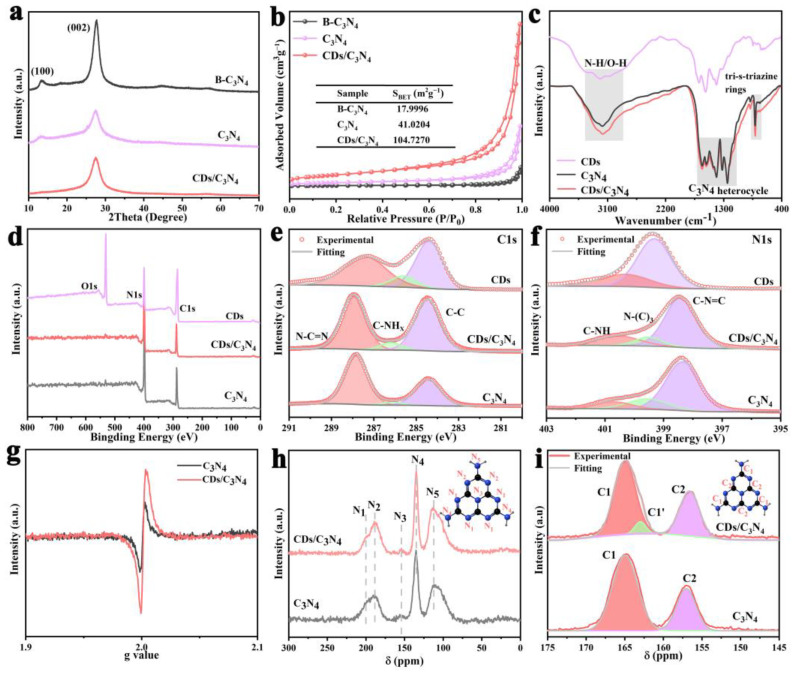
(**a**) XRD patterns of CDs/C_3_N_4_, C_3_N_4_, B-C_3_N_4_. (**b**) Nitrogen adsorption desorption isotherms of CDs/C_3_N_4_, C_3_N_4_, B-C_3_N_4_; (**c**) FT-IR spectra of CDs, CDs/C_3_N_4_, and C_3_N_4_; (**d**) XPS survey spectrum of CDs, C_3_N_4,_ and CDs/C_3_N_4_. (**e**) C1s and (**f**) N1s XPS spectra of CDs, CDs/C_3_N_4,_ and C_3_N_4_. (**g**) EPR spectra, (**h**) ^15^N and (**i**) ^13^C NMR spectra of C_3_N_4_ and CDs/C_3_N_4._

The structure of the synthesized samples is further detected using X-ray diffraction (XRD) and nitrogen adsorption–desorption measurements. For XRD analysis, B-C_3_N_4_ exhibited characteristic diffraction peaks at 2θ = 12.7° and 27.8°, corresponding to the (100) and (002) planes of C_3_N_4_, which represent the structural stacking of tris-triazine (heptazinium) units and the layered assembly of C_3_N_4_ flakes (Figure 2a) [26]. Notably, the progressive weakening and disappearance of the (100) peak of CDs/C_3_N_4_ suggest an alteration of the in-plane aromatic structure [8,27], attributable to the hollow porous structure of the synthesized C_3_N_4_. Nitrogen adsorption–desorption isotherms and the specific surface area of B-C_3_N_4_, C_3_N_4_, and CDs/C_3_N_4_ are displayed in Figure 2b. It can be seen that CDs/C_3_N_4_ possessed a significantly large specific surface area (104.73 m^2^ g^−1^), which is approximately higher than those of B-C_3_N_4_ (17.9996 m^2^ g^−1^) and C_3_N_4_ (41.02 m^2^ g^−1^). This increase, consistent with SEM observations, suggests the successful synthesis of C_3_N_4_ with an enhanced porous structure.

The structural changes before and after CD loading can be revealed by using Fourier transform infrared spectroscopy (FT-IR) and X-ray photoelectron spectroscopy (XPS). Specifically, the FT-IR spectrum of C_3_N_4_ shows peaks within the range of 1200–1700 cm^−1^ and 700–900 cm^−1^, which can be attributed to the stretching mode of the CN heterocyclic ring and tri-s-triazine rings inherent to the C_3_N_4_ structure (Figure 2c). Additionally, a broad absorption band is noted from 3000 to 3500 cm^−1^, associated with terminal amino groups with the N-H component and hydroxyl groups of C_3_N_4_. For the CDs, the stronger peak at around 3100 cm^−1^ suggests a high presence of amino groups in the structure of CDs. Furthermore, the FT-IR analysis of the CDs/C_3_N_4_ indicates that the composite not only retains the characteristic structural features of C_3_N_4_ but also incorporates additional functional groups from CDs. It can be seen that the peak at around 3100 cm^−1^ is stronger than that of the pure C_3_N_4_, which reveals that the composite contains more terminal amine groups. This indicates the successful integration of the CDs into the composite. Furthermore, the enhanced presence of terminal amine groups in the CDs-modified material suggests improved potential for subsequent coordination and anchoring of a Pt cocatalyst. XPS spectra also present the enhanced amount of terminal amine groups after loading CDs onto C_3_N_4_ (Figure 2d). The high-resolution C1s spectra of CDs/C_3_N_4_ exhibit three distinct peaks at 284.54, 286.24, and 288.02 eV, corresponding to C-C, C-NH_x_, and N-C=N bonds (Figure 2e, Table S1) [10,12]. It can be seen that the area ratio of C-NH_x_ bonds in CDs/C_3_N_4_ is significantly enhanced compared to C_3_N_4_. Additionally, from the high-resolution N1s spectra, three distinct peaks at 284.54, 286.24, and 288.02 eV can be attributed to C-NH, N-(C)_3_, and C-N=C bonds. CDs/C_3_N_4_ also exhibit an enhanced area ratio of C-NH groups (Figure 2f, Table S2) [12]. The enhanced amount of terminal amine groups is beneficial for the subsequent loading of the Pt cocatalyst.

Electron paramagnetic resonance (EPR) spectroscopy and solid-state nuclear magnetic resonance (NMR) analyses prove the formation of nitrogen vacancies in CDs/C_3_N_4_. As shown in Figure 2g, the intensity of the EPR signal of CDs/C_3_N_4_ at the g-factor of 2.003 shows an obvious increase in C_3_N_4_, thus indicating the enhanced amount of nitrogen vacancies [9]. ^13^C and ^15^N NMR were carried out to further prove the nitrogen vacancies’ amount (Figure 2h,i). The ^15^N NMR spectrum of C_3_N_4_ reveals signals at the chemical shifts of 199.5 ppm, 187.9 ppm, 153.6 ppm, 134.4 ppm, and 111.7 ppm, which are associated with different nitrogen environments: pyridinic nitrogen adjacent to NH groups (N1), terminal NH_2_ (N2), the heptazine core’s central nitrogen (N3), -NH-bridging nitrogen within the polymer chain (N4), and non-hydrogen-bonded -NH_2_ (N5), respectively [26]. After the introduction of CDs with the solvent-assistant reflux stripping strategy, the N2/N1 area ratio of CDs/C_3_N_4_ (5.9) becomes higher than that of C_3_N_4_ (2.1) (Figure 2h and Figure S6), suggesting the formation of more pyridinic nitrogen vacancies in CDs/C_3_N_4_. ^13^C NMR spectra were further collected with strong resonance peaks at 165.6 ppm and 156.7 ppm, belonging to CN_2_NH_X_ (C1) and C-N_3_ (C2) [26,28], respectively. It should be noted that, in the ^13^C spectra of CDs/C_3_N_4_, an additional peak at 163.1 ppm appears (Figure 2i), which indicates the formation of nitrogen vacancies changes the charge distribution in the heptazine rings, leading to an increased electron cloud density by the transfer of electrons from the vacancies to C1 [28,29,30]. The increased amount of nitrogen vacancies introduces localized states in the band structure of C_3_N_4_, acting as traps for photo-generated electrons and holes. This trapping effect can prolong the lifetime of charge carriers, reducing their recombination rate [9]. As a result, more electrons and holes can participate in the photocatalytic reaction, improving hydrogen production rates.

### 2.2. Band Gap and Photogenerated Charge Transfer

**Figure 3:**
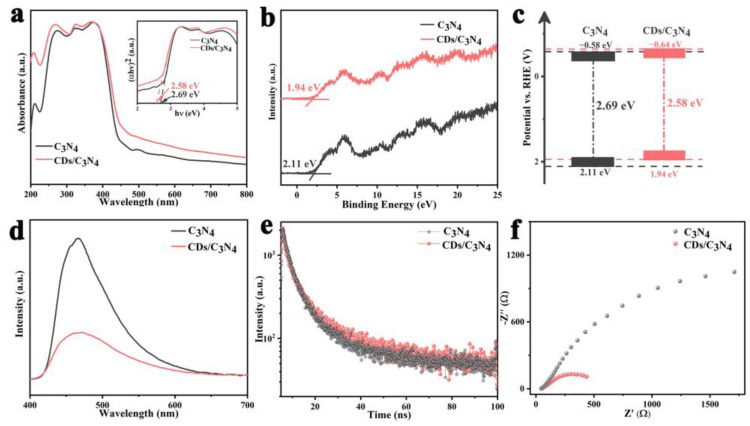
(**a**) UV−vis absorption spectra and (inset) corresponding band gap energies of C_3_N_4_ and CDs/C_3_N_4_; (**b**) XPS valence band for C_3_N_4_ and CDs/C_3_N_4_; (**c**) energy band diagrams of C_3_N_4_ and CDs/C_3_N_4_; (**d**) steady-state PL spectra (365 nmexcitation); (**e**) transient state photoluminescence spectra of C_3_N_4_ and CDs/C_3_N_4_; (**f**) EIS Nyquist plots of C_3_N_4_ and CDs/C_3_N_4_ electrodes.

The band gaps of the synthesized samples were probed using UV-vis diffuse reflectance spectroscopy. It reveals a notable extension in the absorption range of CDs/C_3_N_4_ compared to C_3_N_4_ (Figure 3a), indicating a broadening of light absorption in the visible regions. This suggests that the incorporation of CDs into C_3_N_4_ significantly enhances the adsorption of visible light. A reduction in the band gap, from 2.69 eV in C_3_N_4_ to 2.58 eV in CDs/C_3_N_4_ (inset in Figure 3a), can also be observed. This reduction lowers the energy required for electron excitation, thereby widening the light absorption spectrum. The valence band XPS spectra further illuminate this change, showing a lower VB potential in CDs/C_3_N_4_ (1.94 eV) compared to C_3_N_4_ (2.11 eV) (Figure 3b). The band structures of C_3_N_4_ and CDs/C_3_N_4_ are depicted in Figure 3c. Note that the CDs/C_3_N_4_ possesses a more negative CB as compared to the C_3_N_4_, indicating that the CDs/C_3_N_4_ is thermodynamically more favorable for proton reduction. The introduction of CDs not only affects the visible light utilization of the material but also greatly inhibits the recombination of photogenerated carriers.

To explore the charge dynamics within these materials, steady-state and time-resolved photoluminescence (PL) spectroscopy were employed. The steady-state PL spectra (Figure 3d) showed a significantly reduced peak intensity for CDs/C_3_N_4_ relative to C_3_N_4_, signaling an effective suppression in the electron–hole recombination rate [31]. This is a desirable trait for photocatalysts as it allows for more efficient utilization of photogenerated carriers. Furthermore, time-resolved PL results indicated longer radiative recombination lifetimes in CDs/C_3_N_4_ (10.28 ns) than C_3_N_4_ (7.94 ns) (Figure 3e, Table S3), affirming the enhanced charge separation efficiency imparted by the CDs. The electrical properties of the composites were evaluated using electrochemical impedance spectroscopy (EIS). The Nyquist plots demonstrate that CDs/C_3_N_4_ possesses the best charge-transfer ability with lower charge-transfer resistance and faster electron-transfer kinetics (Figure 3f) [32], which indicates that the CDs/C_3_N_4_ has greatly promoted the charge migration and separation rate [33].

This is further validated by transient photocurrent measurements (Figure S7a). The CDs/C_3_N_4_ aerogel shows an obvious enhancement of photocurrent compared with C_3_N_4_, indicating an excellent light response-ability and greatly enhanced charge separation. Additionally, linear scanning voltammetry (LSV) results revealed that CDs/C_3_N_4_ achieved higher current densities under identical voltage conditions, implying a greater density of photogenerated carriers (Figure S7b). These combined observations underscore CDs/C_3_N_4_ as a potent photocatalyst, with its superior charge-carrier generation and transfer capabilities under light irradiation setting the stage for remarkable hydrogen evolution performance.

### 2.3. Anchoring Effect of CDs on Pt Cocatalyst

**Figure 4:**
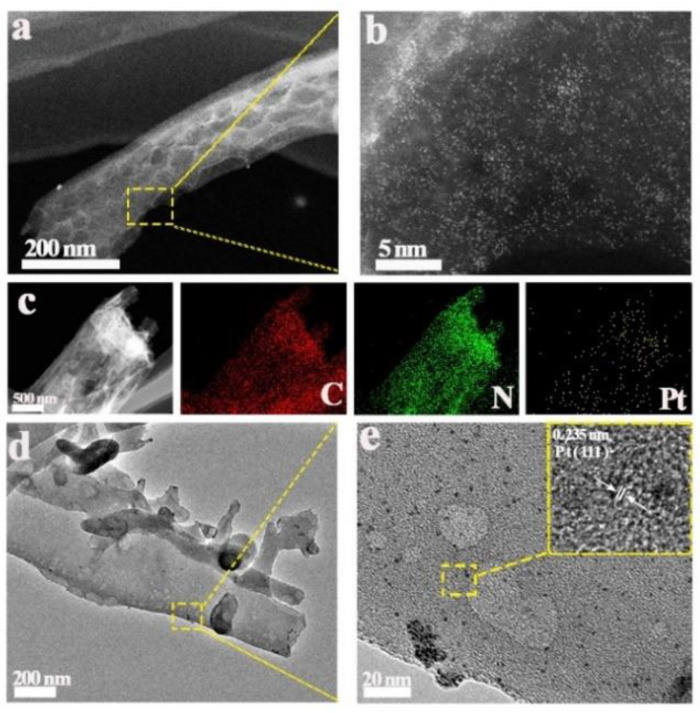
(**a**,**b**) Representative HAADF-STEM image of Pt-CDs/C_3_N_4_; (**c**) the corresponding EDX maps of Pt-CDs/C_3_N_4_. (**d**,**e**) TEM and HRTEM image of the Pt-C_3_N_4_.

The introduction of CDs in the CDs/C_3_N_4_ composite also has a significant anchoring effect on the Pt co-catalyst. The high-resolution, high-angle annular dark-field (HAADF) image reveals highly dispersed Pt atoms (bright spots) uniformly embedded within the porous CDs/C_3_N_4_ (Figure 4a,b). This uniform distribution is further evidenced by the energy-dispersive X-ray spectroscopy (EDS) mapping of Pt-CDs/C_3_N_4_, which shows the high dispersion of Pt, N, and C elements (Figure 4c). In contrast, TEM images of Pt/C_3_N_4_ without the addition of CDs clearly exhibit larger, randomly distributed Pt nanoparticles on the surface of the C_3_N_4_ with a particle size of about 1.2 nm (Figure 4d,e and Figure S8), and the lattice fringe of 0.235 nm corresponds to the (111) lattice plane of Pt nanoparticles [34,35]. The change in Pt particle sizes is mainly due to the introduction of CDs, which can effectively anchor small-sized Pt metal onto C_3_N_4_.

**Figure 5:**
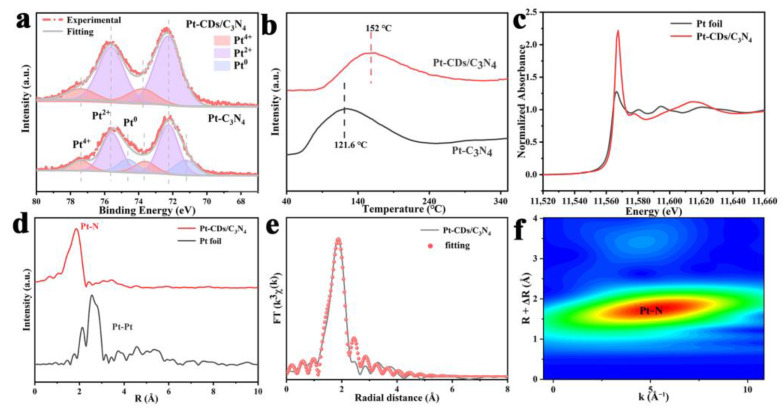
(**a**) High-resolution XPS spectra of Pt 4f for Pt-C_3_N_4_ and Pt-CDs/C_3_N_4_, (**b**) H_2_-TPD spectra of Pt-C_3_N_4_ and Pt-CDs/C_3_N_4_, (**c**) Pt L3-edge XANES spectra, (**d**) FT-EXAFS Pt curves of the Pt Foil and Pt CDs/C_3_N_4_, (**e**) the Fourier-transform EXAFS R-space fitting curve of Pt-CDs/C_3_N_4_, and (**f**) Wavelet transformed k^2^-weighted EXAFS spectra of Pt foil.

Further, the X-ray photoelectron spectroscopy (XPS) analysis of the Pt 4f region provides deeper insights into the anchoring effect of single-atom Pt on CDs (Figure 5a, Table 1). For the Pt 4f spectrum of Pt-C_3_N_4_, six distinct peaks can be found, including the doublet peaks at 72.23 eV and 75.62 eV, attributed to the 4f_7/2_ and 4f_5/2_ states of Pt^2+^, the doublet peaks at 73.74 eV and 77.38 eV, attributed to those of Pt^4+^; and the double peaks at 71.20 eV and 74.63 eV, attributed to metallic Pt^0^ species [36]. The detailed content of the Pt oxidation state and percentage is shown in Table 1. It can be seen that the existence of Pt^0^ with a content of 19.2% in Pt/C_3_N_4_, which indicates the presence of Pt nanoparticles, is in agreement with the TEM result. But for Pt-CDs/C_3_N_4_, there are only four distinct peaks belonging to Pt^2+^ and Pt^4+^, without any Pt^0^ (Figure 5a and Table 1). It suggests that the Pt is anchored on the CDs/C_3_N_4_ in the form of single atoms. H_2_-temperature programmed desorption (H_2_-TPD) (Figure 5b) further reveals the interaction between Pt species and C_3_N_4_. The reduction temperature of Pt species in Pt-CDs/C_3_N_4_ (152 °C) is higher than that of Pt-C_3_N_4_ (121.6 °C), demonstrating that the interaction between Pt species and CDs/C_3_N_4_ is stronger than C_3_N_4_, which greatly stabilizes the Pt species to form the single-atom structures [37,38].

To further explore the chemical state and coordination environment of the single-atom Pt in Pt-CDs/C_3_N_4_, X-ray absorption spectroscopy (XAS) was conducted (Figure 5c). The XANES spectra show a significantly higher white-line intensity compared to Pt foil, indicating the coexistence of Pt^2+^ and Pt^4+^ states [39]. Fourier transform (FT) curves of the extended X-ray absorption fine structure (EXAFS) are plotted to analyze the local atomic structure of the single-atom Pt. The obtained r-space fitting curves and parameters are shown in Figure 5e and Table S4. Different from the Pt-Pt connection in metallic Pt with the main peak at ~2.57 Å [40], it can be seen that the main peak of Pt-CDs/C_3_N_4_ centered at ~1.83 Å (Figure 5d), which is attributed to the Pt-N coordination with an average bond length of 2.10 Å [41]. The coordination number of the Pt-N coordination is 5 [42]. Wavelet transform (WT) analysis in the k-space of EXAFS at the Pt L3 edge of Pt-CDs/C_3_N_4_ (Figure 5f) also shows the Pt-N connection with the center at around ~5.0 Å*^−^*^1^. As the maximum of the WT intensity associated with the Pt-Pt coordination is at ~8.5 Å*^−^*^1^, these results indicate that no Pt nanoparticles are present in Pt-CDs/C_3_N_4_. Therefore, the introduction of CDs into the composite leads to a unique interaction between the Pt cocatalyst and the C_3_N_4_. CDs act as a stabilizing force, preventing the agglomeration of Pt species, thus ensuring a single-atom dispersion. The improved interactions between Pt species and CDs/C_3_N_4_ with Pt-N connection can effectively raise the charge separation efficiency under light excitation to promote hydrogen evolution.

### 2.4. Enhanced Photocatalytic Performance of Pt-CDs/C_3_N_4_

**Figure 6:**
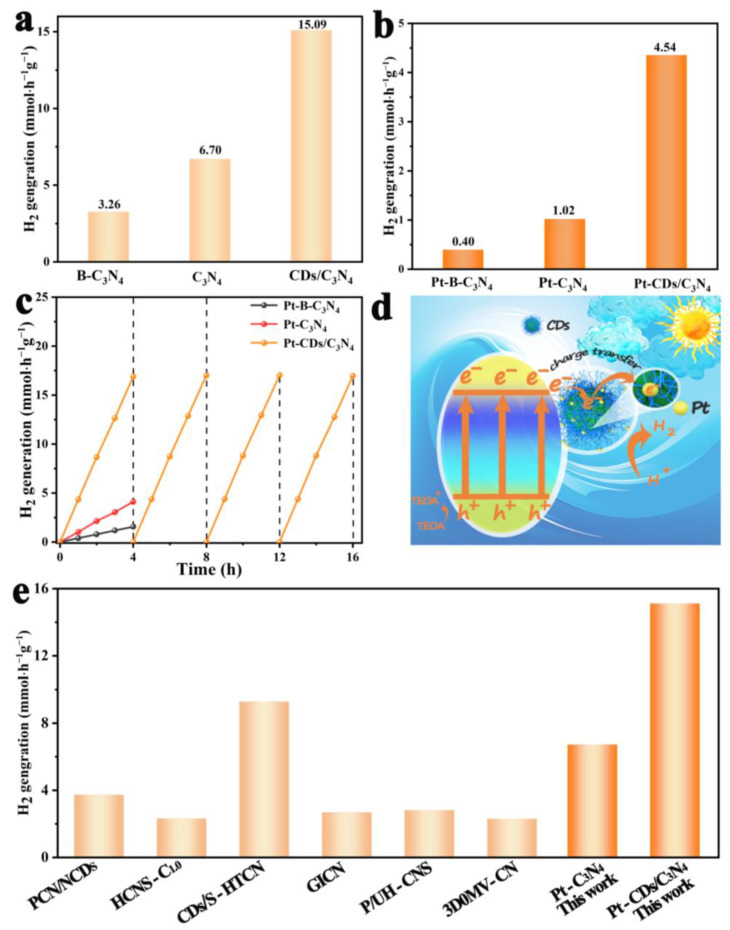
(**a**) Photocatalytic H_2_ evolution rate for Pt-CDs/C_3_N_4_, Pt-C_3_N_4_, Pt-B-C_3_N_4_ using AM 1.5 illumination, (**b**)Photocatalytic H_2_ evolution rate for Pt-CDs/C_3_N_4_, Pt-C_3_N_4_, Pt-B-C_3_N_4_ using CUT 420 illumination. (**c**) The recycled photocatalytic measurement of CDs/C_3_N_4_ using CUT 420 illumination for H_2_ evolution, each of the experiments lasted 4 h, and then the catalyst was recycled and used for the next 4 h cycle, (**d**) Charge transfer and separation mechanism of CDs/C_3_N_4_ under visible light irradiation, (**e**) Photocatalytic hydrogen evolution performance for Pt-CDs/C_3_N_4_ composite in comparison with other CN-based photocatalysts (PCN/NCDs [20], HCNS-C_1.0_ [43], CDs/S-HTCN [44], GICN [11], P/UH-CNS [45], 3D0MV-CN [9]) that reported in recent years [9,11,20,43,44,45].

The integration of CDs into the filamentary C_3_N_4_ composite significantly elevates its photocatalytic performance. After photo-deposition of Pt cocatalyst, Pt-CDs/C_3_N_4_ demonstrates exceptional hydrogen evolution performance under visible light irradiation, achieving a rate of 15.09 mmol h*^−^*^1^ g*^−^*^1^, which is approximately 4.6 times higher than that of Pt-B-C_3_N_4_ (3.26 mmol h*^−^*^1^ g*^−^*^1^) and 2.3 times higher than that of Pt-C_3_N_4_ (6.70 mmol h*^−^*^1^ g*^−^*^1^) (Figure 6a). Under visible light (λ > 420 nm) irradiation, as presented in Figure 6b, the average hydrogen evolution rate of the Pt-CDs/C_3_N_4_ achieves 4.54 mmol h*^−^*^1^ g*^−^*^1^, which is about 11.3 times higher than that of Pt-B-C_3_N_4_ (0.40 mmol h*^−^*^1^ g*^−^*^1^) and 4.45 times higher than that of Pt-C_3_N_4_ (1.02 mmol h*^−^*^1^ g*^−^*^1^), respectively. Moreover, there is no noticeable attenuation in the H_2_ production rate after four cycling tests within the 16 h photocatalytic period (Figure 6c), suggesting the high stability of Pt-CDs/C_3_N_4_ under the reaction conditions. The reaction quantum yields of Pt-C_3_N_4_ and Pt-CDs/C_3_N_4_ under 420 nm light irradiation were calculated to be 2.4% and 4.5%, respectively. The promoted quantum yields of Pt-CDs/C_3_N_4_ should be due to the improved charge separation efficiency and enhanced light absorption with the introduction of CDs. Meanwhile, the hydrogen evolution performance of 5-Pt-CDs/C_3_N_4_ and 15-Pt-CDs/C_3_N_4_ materials in the visible region are 3.12 mmol h*^−^*^1^ g*^−^*^1^ and 3.66 mmol h*^−^*^1^ g*^−^*^1^, respectively (Figure S10), which shows that the loading content of CDs also has a significant effect on the properties. As shown in Figure 6e, Pt-CDs/C_3_N_4_ exhibits excellent hydrogen evolution performance compared to the state-of-the-art CN-based photocatalysts. The enhanced hydrogen evolution performance should be attributed to the introduction of CDs in the C_3_N_4_ matrix and their anchoring effect on Pt. With the loading of CDs, Pt-CDs/C_3_N_4_ photocatalyst shows enhanced spectral response and abundant active site, as well as fast charge separation efficiency and activation energy level for the strong photocatalytic hydrogen production performance. The rich functional groups on the surface of CDs can effectively stabilize single-atom Pt cocatalysts, thereby increasing the contact efficiency with reactants. Meanwhile, strong interactions via Pt-N bonds can enhance the efficiency of photogenerated electron separation. These unique properties can collectively enhance photocatalytic hydrogen evolution efficiency.

## 3. Experimental

### 3.1. Materials

Citric acid, urea, melamine, phosphoric acid, ethanol, glycerol, chloroplatinic acid hexahydrate (H_2_PtCl_6_·6H_2_O), triethanolamine, and sodium sulfate. All chemicals were used as received without any further purification. The water used in all experiments was deionized water.

### 3.2. Synthesis of Carbon Dots

The CDs were obtained by a modified hydrothermal method according to previous literature, where 3 g of citric acid was dissolved in 20 mL of water and 1 g of urea [22]. The mixture was then autoclaved in a Teflon container at 180 °C for 5 h. The dark brown solution obtained was centrifuged at high speed (10,000 rad min^−1^) for 20 min to remove large or agglomerated particles. The final products were obtained by lyophilization.

### 3.3. Synthesis of Precursor

As previously reported in the literature [12], 1 g of melamine and 1.2 g of phosphoric acid were dissolved in 100 mL of deionized water and stirred vigorously for 1 h at 80 °C in a constant-temperature water bath. The solution was then transferred to a Teflon autoclave and heated at 180 °C for 10 h. The mixture was centrifuged and dried at 60 °C to obtain the precursor.

### 3.4. Synthesis of Hollow Porous CDs/C_3_N_4_

An amount of 0.6 g precursors were refluxed with a mixed aqueous solution of 5 mL glycerol, 15 mL ethanol, and 10 mg CDs for 3 h at 90 °C. The powders were then washed three times with ethanol and dried at 60 °C. Finally, the resulting solids were heated to 500 °C in a muffle furnace at a heating rate of 2 °C/min and held for 2 h. The sample was named CDs/C_3_N_4_. Different CDs/C_3_N_4_ materials were prepared by varying the mass of CDs (5 mg, 15 mg), which were named 5-CDs/C_3_N_4_ and 15-CDs/C_3_N_4_, respectively.

### 3.5. Synthesis of Pt-C_3_N_4_ and Pt-CDs/C_3_N_4_

An amount of 10 mg of C_3_N_4_ and CDs/C_3_N_4_ were dispersed in 10 mL water, followed by the addition of chloroplatinic acid (0.004 g/mL, 0.07 mL) to the solution. Then, photo-deposition was carried out under a 300 W xenon lamp with an AM 1.5 filter for 40 min, followed by freeze-drying the samples to obtain the Pt-C_3_N_4_ and Pt-CDs/C_3_N_4_ composites. The target loading content of Pt was 1 wt%.

### 3.6. Synthesis of C_3_N_4_

The precursors were heated to 500 °C in a muffle furnace at a heating rate of 2 °C/min and held for 2 h. The resulting material was named C_3_N_4_.

### 3.7. Synthesis of B-C_3_N_4_

An amount of 5 g of melamine was placed in a porcelain crucible and calcined at 500 °C in a muffle furnace for 4 h. The product obtained was ground to a homogeneous powder and named B**-**C_3_N_4_.

### 3.8. Characterization of Sample

X-ray powder diffraction (XRD) patterns were obtained by Bruker D8. Scanning electron microscopy (SEM, S-4800, Hitachi, Tokyo, Japan), transmission electron microscopy (TEM) images, high-resolution TEM images (HRTEM, Technai G2 F20, FEI, USA), and aberration-corrected high-angle annular dark-field scanning TEM images (AC-HAADF-STEM, TALOS F200X microscope) (Thermo Scientific, Waltham, MA, USA) were adopted to observe the morphologies of the samples. Fourier transform infrared (FT-IR) spectroscopy was recorded on a Nicolet is 50 FT-IR spectrometer (Thermo Scientific, Waltham, MA, USA), using KBr as the diluent. X-ray photoelectron spectroscopy (XPS) analysis was performed on a VG ESCALAB MK II (VG, UK) with an Mg Kα (1253.6 eV) achromatic X-ray source. N_2_ adsorption–desorption isotherms at 77 K were collected using a Micromeritics ASAP2420 (Micromeritics, USA) surface area and porosity analyzer. Before the measurement, all the samples were degassed under a vacuum at 150 °C for 10 h. The Brunauer–Emmett–Teller (BET) equation method was used to calculate the total surface area. ^13^C MAS NMR spectra were collected using a Bruker Avance-300 spectrometer with a 4 mm zirconia rotor. UV−vis absorption spectra were determined at room temperature on a spectrophotometer (UV-2700, Shimadzu, Japan). Electron paramagnetic resonance (EPR) measurements were performed on a Bruker EMX plus model spectrometer (Bruker, Karlsruhe, German). The photoluminescence (PL) spectra of the photocatalysts were measured on an Edinburgh instrument FLS920 fluorescence spectrophotometer (Edinburgh, Livingston, UK). The H_2_-temperature-programmed desorption (H_2_-TPD) was detected by an automatic chemical adsorption instrument (FINETEC/FINE-SORB-3010, Finetec, Quzhou, China).

### 3.9. Photocatalytic H_2_ Production Experiments

The photocatalytic hydrogen production experiments were performed in an online photocatalytic hydrogen production (Labsolar 6A) (Pophilet, Beijing, China), connected to an online gas chromatograph (GC7900, TCD) (Tianmei, Shanghai, China) using argon gas as the carrier gas. The photocatalyst (10 mg) was dispersed in a mixture of 80 mL distilled water and 20 mL triethanolamine in the reaction cell by using a magnetic stirrer (MS5, JOANLAB, Huzhou, China). Prior to the reaction, the mixture was degassed under vacuum to remove O_2_ and CO_2_. The photocatalytic reaction is conducted under a xenon lamp (300 W) with filters (CUT 420 and AM 1.5) as a light source. The light intensities were 0.38 W/cm^2^ and 0.29 W/cm^2^ with AM 1.5 and CUT 420 filters, respectively. The temperature of the reaction system was controlled at 20 °C by injecting constant-temperature water into the reactor sandwich. The evolution gas was analyzed by an on-line gas chromatograph (GC7900, TCD).

The determination of the apparent quantum efficiency (AQE) for hydrogen generation was performed using the closed system under the illumination of a 300 W xenon lamp with 420 nm bandpass filters. The AQE was calculated by using the following equation:

$$\mathrm{AQE}=\frac{2\;N(H_{2})}{N\;(photons)}\times 100\%\;=\frac{2\times n\left(H_{2}\right)\times N_{A}\times h\times c}{F\times S\times T\times \lambda }\;\times \;100\%$$

where N(H_2_) is the number of evolved H_2_ molecules, N (Photons) is the number of incident photons, n(H_2_) refers to the hydrogen evolution (mol), T is the irradiation time (s), N_A_ is the Avogadro constant (6.022 × 10^23^ mol^−l^), F refers to the average spectral irradiance (W/cm^2^), S is the irradiation area (23.74 cm^2^), λ is the wavelength of monochromatic light (m), h is the Planck constant (6.626 × 10^−34^ J·s) and c is the light speed (3.0 × 10^8^ m·s^−1^).

### 3.10. Photoelectrochemical Measurements

The photochemical tests of the samples were performed in a conventional three-electrode quartz cell using a computer-controlled CHI660E electrochemical workstation (Chenhua, Shanghai, China). The light source was a 300 W xenon lamp with a simulated sunlight filter (AM 1.5). An Ag/AgCl electrode and a platinum wire were used as the reference electrode and the counter electrode, respectively. A Na_2_SO_4_ solution (0.2 mol L^−1^) was used as the electrolyte after nitrogen was injected for 3 h. The process to prepare the working electrode with the as-obtained sample was as follows: 10 mg of catalyst was dispersed in 2 mL of ethanol. After ultrasonic treatment, the obtained homogeneous suspension was sprayed on the surface of fluorine-doped tin oxide glass with an area of 1 cm^2^ and calcined at 350 °C for 2 h in a nitrogen atmosphere for further use.

## 4. Conclusions

In conclusion, our research has successfully developed a novel photocatalyst, Pt-CDs/C_3_N_4_, by synergistically combining single-atom Pt cocatalysts with a CD-enhanced C_3_N_4_. This approach, leveraging the anchoring effect of CDs and the enhanced porous structure of C_3_N_4_, not only improves the light absorption capabilities but also enhances active site availability and charge separation efficiency. The integration of -NH_2_ functional groups on CDs effectively anchors single-atom Pt, facilitating strong Pt-N bond formations. This unique structural feature greatly improves the efficiency of photogenerated electron separation, resulting in a remarkable hydrogen evolution rate of 15.09 mmol h^−1^ g^−1^ under visible light irradiation. This performance is 4.6 times higher than that of Pt-traditional bulk C_3_N_4_, highlighting the exceptional potential of the Pt-CDs/C_3_N_4_ composite for sustainable hydrogen production.

## Figures and Tables

**Table 1:** Type and contents of Pt oxidation state of different samples.

Samples	Type and Contents of Elements (%)		
	Pt		
	Pt^4+^	Pt^2+^	Pt^0^
Pt-C_3_N_4_	17	63.8	19.2
Pt-CDs/C_3_N_4_	16.2	83.8	0

## Data Availability

Data are contained within the article and Supplementary Materials.

## Supplementary Materials

The following supporting information can be downloaded at https://www.mdpi.com/article/10.3390/molecules29081890/s1. Figure S1: SEM image of the 5-CDs/FC_3_N_4_. Figure S2: SEM image of the 15-CDs/FC_3_N_4_. Figure S3: SEM image of the B-C_3_N_4_. Figure S4: SEM image of the C_3_N_4_. Figure S5: XRD patterns of 5-CDs/C_3_N_4_, 15-CDs/C_3_N_4_, CDs/C_3_N_4_. Figure S6: Peak-differentiating analysis of the N1 and N2 peaks in the ^15^N NMR spectra of CDs/C_3_N_4_and C_3_N_4_. Figure S7: (a) Transient photocurrent response of C_3_N_4_ and CDs/C_3_N_4_ with repeated on-off cycles under simulated sunlight irradiation, (b) Linear sweeps voltammograms of C_3_N_4_ and CDs/C_3_N_4_. Figure S8: The particle size distribution map of Pt nanoparticles on C_3_N_4_. Figure S9: XPS survey spectrum of Pt-C_3_N_4_ and Pt-CDs/C_3_N_4_. Figure S10: Photocatalytic H_2_ evolution rate for 5-CDs/C_3_N_4_ and 15-CDs/C_3_N_4_. Table S1: Type and contents of elements of different samples. Table S2: Type and contents of elements of different samples. Table S3: PL lifetime of photogenerated charge carrier. Table S4: EXAFS fitting parameters at the Pt L3-edge for various samples (S_0_^2^ = 0.925). Table S5: Photocatalytic hydrogen evolution performance for Pt-CDs/C_3_N_4_ composite in comparison with other CN-based photocatalysts that reported in recent years.

## References

[1] Yuan H., Sun H., Shi Y., Wang J., Bian A., Hu Y., Guo F., Shi W., Du X., Kang Z. (2023). Cooperation of carbon doping and carbon loading boosts photocatalytic activity by the optimum photo-induced electron trapping and interfacial charge transfer. Chem. Eng. J..

[2] Gao G., Niu X., Xu B., Wang X.L., Yao Y.-F. (2020). Shape and size effects on photocatalytic hydrogen production via Pd/C_3_N_4_ photocatalysts under visible light. Catal. Sci. Technol..

[3] Zhai B., Li H., Gao G., Wang Y., Niu P., Wang S., Li L. (2022). A Crystalline Carbon Nitride Based Near-Infrared Active Photocatalyst. Adv. Funct. Mater..

[4] Lu X., Xu K., Chen P., Jia K., Liu S., Wu C. (2014). Facile one step method realizing scalable production of g-C_3_N_4_ nanosheets and study of their photocatalytic H_2_ evolution activity. J. Mater. Chem. A.

[5] Zeng W., Dong Y., Ye X., Guan X., Zhang T., Guo L. (2023). Ultrathin porous carbon nitride with molecular structure regulation for excellent photocatalytic water splitting. Chem. Eng. J..

[6] Zhang M., Wen J., Zhang S., Zhai Y. (2021). Tremella-like porous carbon nitride co-doped with oxygen and carbon towards efficient visible-light-driven purification of wastewater. Sep. Purif. Technol..

[7] Bao H., Wang L., Li G., Zhou L., Xu Y., Liu Z., Wu M. (2021). Carrier engineering of carbon nitride boosts visible-light photocatalytic hydrogen evolution. Carbon.

[8] Xiao X., Gao Y., Zhang L., Zhang J., Zhang Q., Li Q., Bao H., Zhou J., Miao S., Chen N. (2020). A Promoted Charge Separation/Transfer System from Cu Single Atoms and C_3_N_4_ Layers for Efficient Photocatalysis. Adv. Mater..

[9] Li Q., Zhang Y., Zeng Y., Ding M. (2023). Ordered porous nitrogen-vacancy carbon nitride for efficient visible-light hydrogen evolution. J. Colloid Interface Sci..

[10] Xiao Y., Guo S., Tian G., Jiang B., Ren Z., Tian C., Li W., Fu H. (2021). Synergetic enhancement of surface reactions and charge separation over holey C_3_N_4_/TiO_2_ 2D heterojunctions. Sci. Bull..

[11] Yu W., Zhang T., Zhao Z. (2020). Garland-like intercalated carbon nitride prepared by an oxalic acid-mediated assembly strategy for highly-efficient visible-light-driven photoredox catalysis. Appl. Catal. B.

[12] Xiao Y., Tian G., Li W., Xie Y., Jiang B., Tian C., Zhao D., Fu H. (2019). Molecule Self-Assembly Synthesis of Porous Few-Layer Carbon Nitride for Highly Efficient Photoredox Catalysis. J. Am. Chem. Soc..

[13] Ran J., Zhang J., Yu J., Jaroniec M., Qiao S.-Z. (2013). Earth-abundant cocatalysts for semiconductor-based photocatalytic water splitting. Chem. Soc. Rev..

[14] Hu Y., Qu Y., Zhou Y., Wang Z., Wang H., Yang B., Yu Z., Wu Y. (2021). Single Pt atom-anchored C3N4: A bridging Pt–N bond boosted electron transfer for highly efficient photocatalytic H_2_ generation. Chem. Eng. J..

[15] Wang G., Zhang T., Yu W., Si R., Liu Y., Zhao Z. (2020). Modulating Location of Single Copper Atoms in Polymeric Carbon Nitride for Enhanced Photoredox Catalysis. ACS Catal..

[16] Li H., Liu R., Lian S., Liu Y., Huang H., Kang Z. (2013). Near-infrared light controlled photocatalytic activity of carbon quantum dots for highly selective oxidation reaction. Nanoscale.

[17] Liu Z., Hou W., Guo H., Wang Z., Wang L., Wu M. (2023). Functional Group Modulation in Carbon Quantum Dots for Accelerating Photocatalytic CO_2_ Reduction. ACS Appl. Mater. Interfaces.

[18] Wang Y., Liu X., Han X., Godin R., Chen J., Zhou W., Jiang C., Thompson J.F., Mustafa K.B., Shevlin S.A. (2020). Unique hole-accepting carbon-dots promoting selective carbon dioxide reduction nearly 100% to methanol by pure water. Nat. Commun..

[19] Wang X., Li X., Ding S., Chen Y., Liu Y., Fang M., Xiao G., Zhu Y. (2021). Constructing ample active sites in nitrogen-doped carbon materials for efficient electrocatalytic carbon dioxide reduction. Nano Energy.

[20] Zhang S., Yang Y., Zhai Y., Wen J., Zhang M., Yu J., Lu S. (2023). A novel P-doped and NCDs loaded g-C_3_N_4_ with enhanced charges separation for photocatalytic hydrogen evolution. Chin. Chem. Lett..

[21] Miao X., Yue X., Ji Z., Shen X., Zhou H., Liu M., Xu K., Zhu J., Zhu G., Kong L. (2018). Nitrogen-doped carbon dots decorated on g-C_3_N_4_/Ag_3_PO_4_ photocatalyst with improved visible light photocatalytic activity and mechanism insight. Appl. Catal. B.

[22] Choi Y., Choi Y., Kwon O.H., Kim B.S. (2018). Carbon Dots: Bottom-Up Syntheses, Properties, and Light-Harvesting Applications. Chem. Asian J..

[23] Luo H., Liu Y., Dimitrov S.D., Steier L., Guo S., Li X., Feng J., Xie F., Fang Y., Sapelkin A. (2020). Pt single-atoms supported on nitrogen-doped carbon dots for highly efficient photocatalytic hydrogen generation. J. Mater. Chem. A.

[24] Yu H., Shi R., Zhao Y., Waterhouse G.I.N., Wu L.Z., Tung C.H., Zhang T. (2016). Smart Utilization of Carbon Dots in Semiconductor Photocatalysis. Adv. Mater..

[25] Chen J., Xiao Y., Wang N., Kang X., Wang D., Wang C., Liu J., Jiang Y., Fu H. (2023). Facile synthesis of a Z-scheme CeO_2_/C_3_N_4_ heterojunction with enhanced charge transfer for CO_2_ photoreduction. Sci. China Mater..

[26] Li Q., Jiao Y., Tang Y., Zhou J., Wu B., Jiang B., Fu H. (2023). Shear Stress Triggers Ultrathin-Nanosheet Carbon Nitride Assembly for Photocatalytic H_2_O_2_ Production Coupled with Selective Alcohol Oxidation. J. Am. Chem. Soc..

[27] Lu J., Shi Y., Chen Z., Sun X., Yuan H., Guo F., Shi W. (2023). Photothermal effect of carbon dots for boosted photothermal-assisted photocatalytic water/seawater splitting into hydrogen. Chem. Eng. J..

[28] An X., Tang Q., Lan H., Liu H., Yu X., Qu J., Lin H., Ye J. (2022). Facilitating Molecular Activation and Proton Feeding by Dual Active Sites on Polymeric Carbon Nitride for Efficient CO_2_ Photoreduction. Angew. Chem.Int. Ed..

[29] Hu Y., Shim Y., Oh J., Park S., Park S., Ishii Y. (2017). Synthesis of ^13^C-, ^15^N-Labeled Graphitic Carbon Nitrides and NMR-Based Evidence of Hydrogen-Bonding Assisted Two-Dimensional Assembly. Chem. Mater..

[30] Li X., Sergeyev I.V., Aussenac F., Masters A.F., Maschmeyer T., Hook J.M. (2018). Dynamic Nuclear Polarization NMR Spectroscopy of Polymeric Carbon Nitride Photocatalysts: Insights into Structural Defects and Reactivity. Angew. Chem. Int. Ed..

[31] Zhao Y., Liu Y., Wang Z., Ma Y., Zhou Y., Shi X., Wu Q., Wang X., Shao M., Huang H. (2021). Carbon nitride assisted 2D conductive metal-organic frameworks composite photocatalyst for efficient visible light-driven H_2_O_2_ production. Appl. Catal. B.

[32] Wang F., Wang Y., Feng Y., Zeng Y., Xie Z., Zhang Q., Su Y., Chen P., Liu Y., Yao K. (2018). Novel ternary photocatalyst of single atom-dispersed silver and carbon quantum dots co-loaded with ultrathin g-C_3_N_4_ for broad spectrum photocatalytic degradation of naproxen. Appl. Catal. B.

[33] Wang Y., Zhao X., Cao D., Wang Y., Zhu Y. (2017). Peroxymonosulfate enhanced visible light photocatalytic degradation bisphenol A by single-atom dispersed Ag mesoporous g-C_3_N_4_ hybrid. Appl. Catal. B.

[34] Wang H., Yin S., Eid K., Li Y., Xu Y., Li X., Xue H., Wang L. (2018). Fabrication of Mesoporous Cage-Bell Pt Nano architectonics as Efficient Catalyst for Oxygen Reduction Reaction. ACS Sustain. Chem. Eng..

[35] Zaman S., Su Y.Q., Dong C.L., Qi R., Huang L., Qin Y., Huang Y.C., Li F.M., You B., Guo W. (2021). Scalable Molten Salt Synthesis of Platinum Alloys Planted in Metal–Nitrogen–Graphene for Efficient Oxygen Reduction. Angew. Chem.Int. Ed..

[36] Ma Y., Zhang X., Cao L., Lu J. (2021). Effects of the morphology and heteroatom doping of CeO_2_ support on the hydrogenation activity of Pt single-atoms. Catal. Sci. Technol..

[37] Ma W., Sun J., Yao S., Wang Y., Chen G., Fan G., Li Y. (2023). Synergistic Interplay of Dual-Active-Sites on Metallic Ni-MOFs Loaded with Pt for Thermal-Photocatalytic Conversion of Atmospheric CO_2_ under Infrared Light Irradiation. Angew. Chem. Int. Ed..

[38] Zhang W., Wang Y., Gu B., Tang Q., Cao Q.-E., Fang W. (2023). Regulating the Interaction within Pd-Cu Dual Metal Sites for Selective Hydrogenation of Furfural Using Ambient H_2_ Pressure. ACS Sustain. Chem. Eng..

[39] Zhang Z., Chen Y., Zhou L., Chen C., Han Z., Zhang B., Wu Q., Yang L., Du L., Bu Y. (2019). The simplest construction of single-site catalysts by the synergism of micropore trapping and nitrogen anchoring. Nat.Commun..

[40] Yan J., Ji Y., Batmunkh M., An P., Zhang J., Fu Y., Jia B., Li Y., Liu S., Ye J. (2020). Breaking Platinum Nanoparticles to Single-Atomic Pt-C_4_ Co-catalysts for Enhanced Solar-to-Hydrogen Conversion. Angew. Chem. Int. Ed..

[41] Chen W., Luo X., Slater T., Zhou Y., Ling S., Bao R., Fernandes J., Shen W.Y. (2020). General synthesis of single atom electrocatalysts via a facile condensation–carbonization process. J. Mater. Chem. A.

[42] Wang N., Mei R., Lin X., Chen L., Yang T., Liu Q., Chen Z. (2023). Cascade Anchoring Strategy for Fabricating High-Loading Pt Single Atoms as Bifunctional Catalysts for Electrocatalytic Hydrogen Evolution and Oxygen Reduction Reactions. ACS Appl. Mater. Interfaces.

[43] Ding Y., Lin Z., Deng J., Liu Y., Zhang L., Wang K., Xu S., Cao S. (2022). Construction of carbon dots modified hollow g-C_3_N_4_ spheres via in situ calcination of cyanamide and glucose for highly enhanced visible light photocatalytic hydrogen evolution. Int. J. Hydrogen Energy.

[44] Yuan H., Shi W., Lu J., Wang J., Shi Y., Guo F., Kang Z. (2023). Dual-channels separated mechanism of photo-generated charges over semiconductor photocatalyst for hydrogen evolution: Interfacial charge transfer and transport dynamics insight. Chem. Eng. J..

[45] Lv S., Ng Y.H., Zhu R., Li S., Wu C., Liu Y., Zhang Y., Jing L., Deng J., Dai H. (2021). Phosphorus vapor assisted preparation of P-doped ultrathin hollow g-C_3_N_4_ sphere for efficient solar-to-hydrogen conversion. Appl. Catal. B.

