# Supplementary material for: Carbon Dots Anchoring Single-Atom Pt on C3N4 Boosting Photocatalytic Hydrogen Evolution
Source: Molecules. 2024 Apr 21;29(8):1890. doi: 10.3390/molecules29081890 (PMC11055151; doi:10.3390/molecules29081890)
Supplement: Supplementary file 1 [file molecules-29-01890-s001.zip › molecules-2965756-supplementary.pdf]

molecules

Supporting Information

Carbon Dots Anchoring Pt Single Atoms on C_3N_4 Boosting Photocatalytic Hydrogen Evolution

Jing Wang, Jiayu Song, Xin Kang, Dongxu Wang, Chungui Tian, Qin Zhang, Hui Zhao, Jiancong Liu *

Key Laboratory of Functional Inorganic Material Chemistry, Ministry of Education of the People's Republic of China, Heilongjiang University, Harbin 150080, China

* Corresponding authors

E-mail addresses: liujiancong@hlju.edu.cn or jiancong@gmail.com

The content of ESI

1. **Figure S1.** SEM image of the 5-CDs/FC_3N_4.
2. **Figure S2.** SEM image of the 15-CDs/FC_3N_4.
3. **Figure S3.** SEM image of the B-C_3N_4.
4. **Figure S4.** SEM image of the C_3N_4.
5. **Figure S5.** XRD patterns of 5-CDs/C_3N_4, 15-CDs/C_3N_4, CDs/C_3N_4.
6. **Figure S6.** Peak-differentiating analysis of the N1 and N2 peaks in the ^{15}N NMR spectra of CDs/$C_3N_4$and C_3N_4.
7. **Figure S7.** (a) Transient photocurrent response of C_3N_4 and CDs/C_3N_4 with repeated on-off cycles under simulated sunlight irradiation, (b) Linear sweeps voltammograms of C_3N_4 and CDs/C_3N_4.
8. **Figure S8.** The particle size distribution map of Pt nanoparticles on C_3N_4.
9. **Figure S9.** XPS survey spectrum of Pt-C_3N_4 and Pt-CDs/C_3N_4.
10. **Figure S10.** Photocatalytic H_2 evolution rate for 5-CDs/C_3N_4 and 15-CDs/C_3N_4.
11. **Table S1.** Type and contents of elements of different samples.
12. **Table S2.** Type and contents of elements of different samples.
13. **Table S3.** PL lifetime of photogenerated charge carrier.
14. **Table S4.** EXAFS fitting parameters at the Pt L3-edge for various samples (S_0^2=0.925).
15. **Table S5.** Photocatalytic hydrogen evolution performance for Pt-CDs/C_3N_4 composite in comparison with other CN-based photocatalysts that reported in recent years.

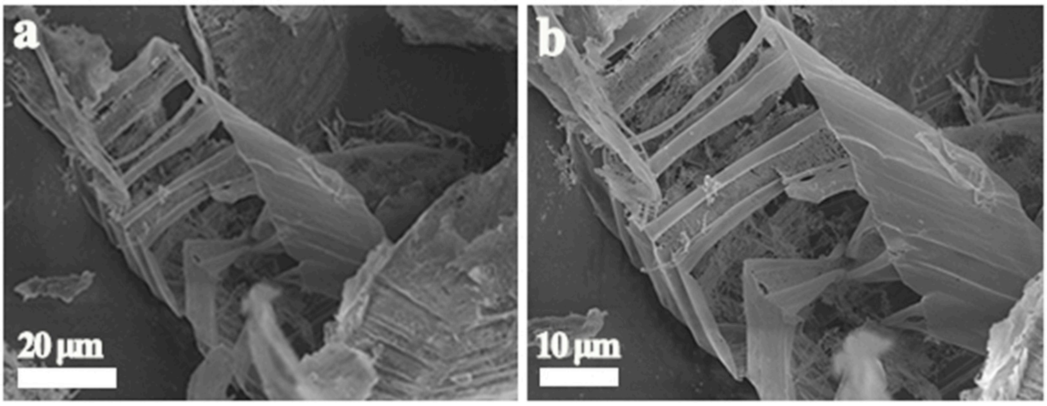

Figure S1. SEM image of the 5-CDs/C_3N_4.

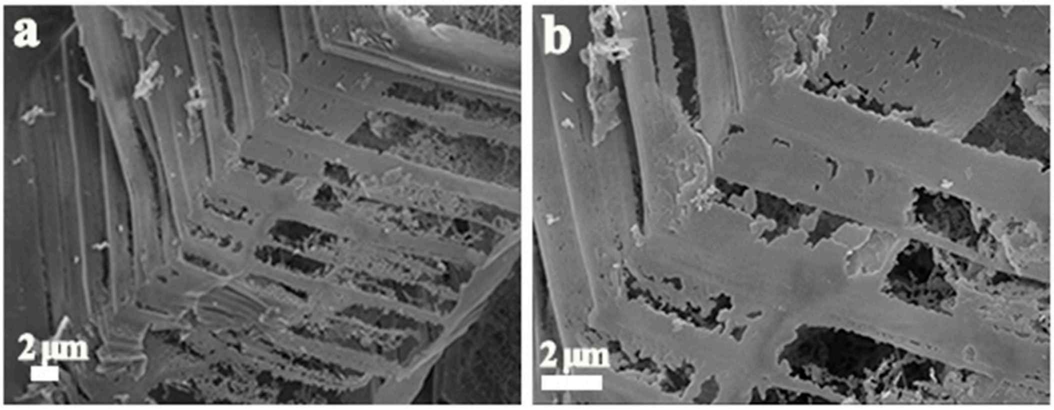

Figure S2. SEM image of the 15-CDs/C_3N_4.

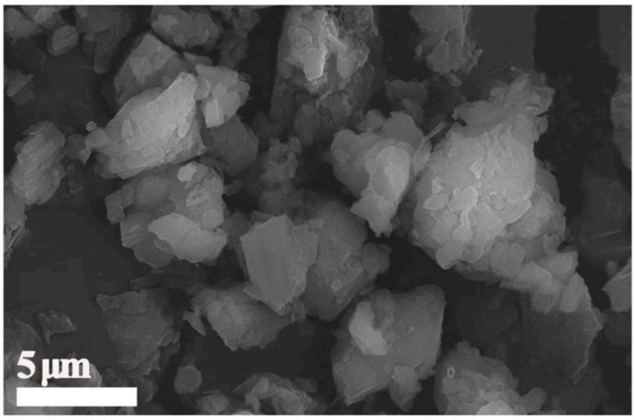

Figure S3. SEM image of the B-C_3N_4.

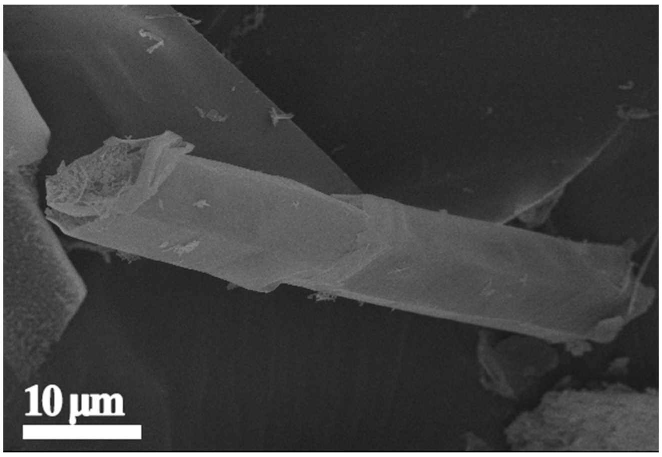

Figure S4. SEM image of the C_3N_4.

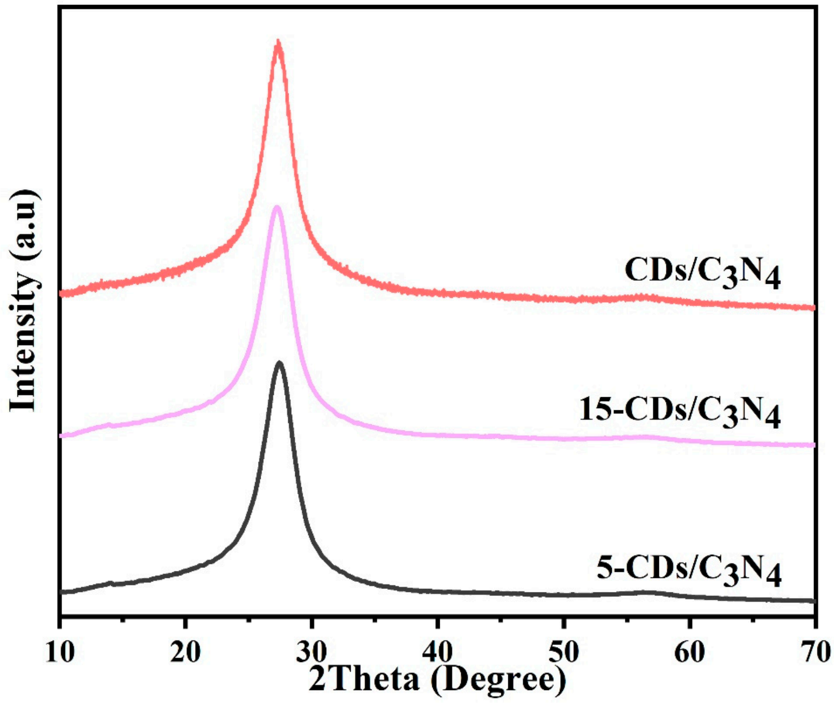

Figure S5. XRD patterns of 5-CDs/ C_3N_4, 15-CDs/ C_3N_4, CDs/C_3N_4.

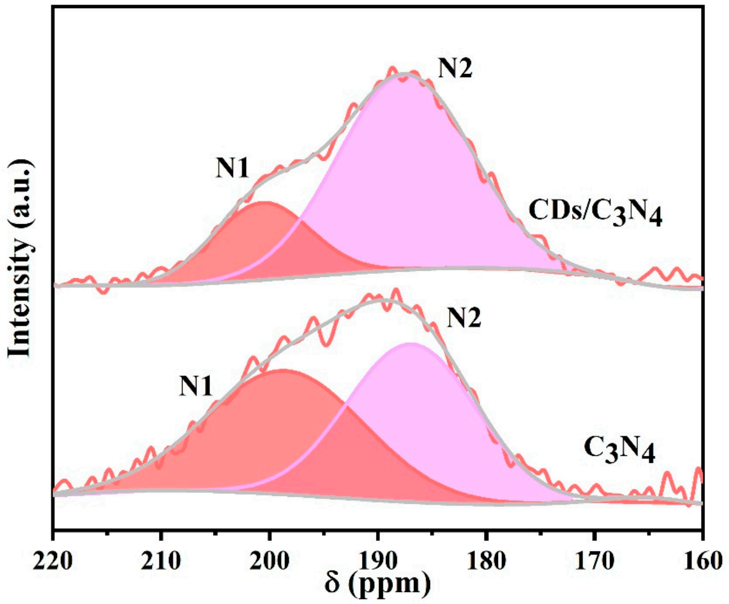

Figure S6. Peak-differentiating analysis of the N1 and N2 peaks in the ^{15}N NMR spectra of CDs/C_3N_4 and C_3N_4.

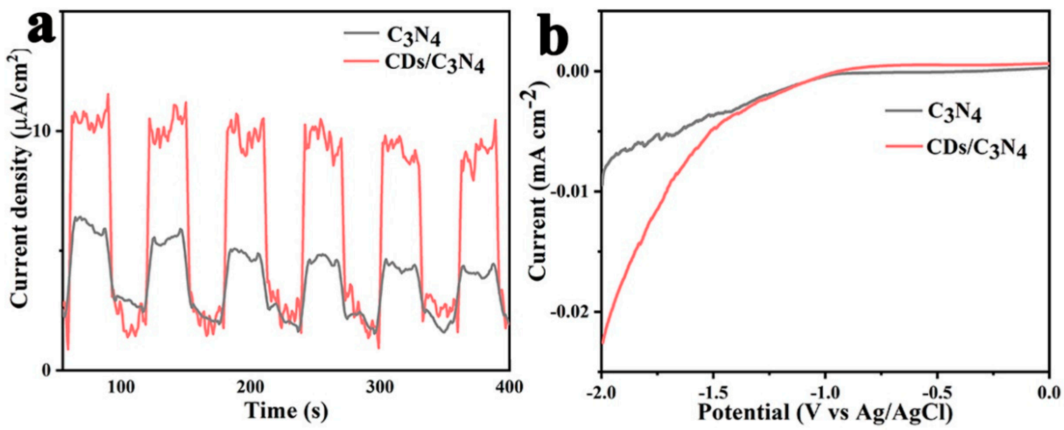

Figure S7. (a) Transient photocurrent response of C_3N_4 and CDs/C_3N_4 with repeated on-off cycles under simulated sunlight irradiation, (b) Linear sweeps voltammograms of C_3N_4 and CDs/C_3N_4.

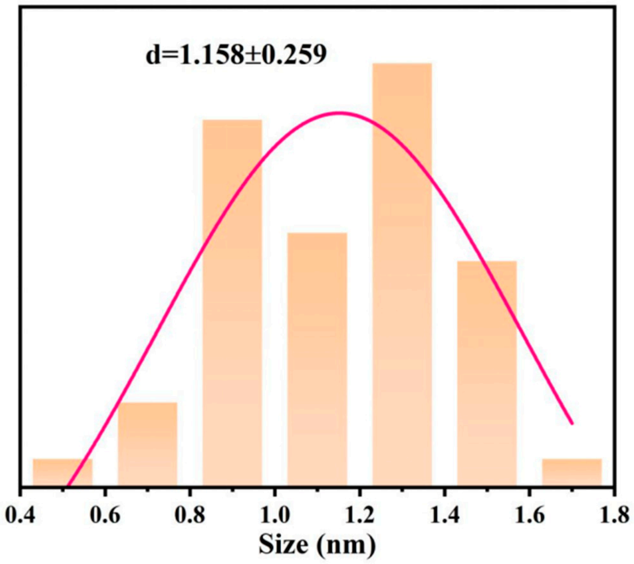

Figure S8. The particle size distribution map of Pt nanoparticles on C_3N_4.

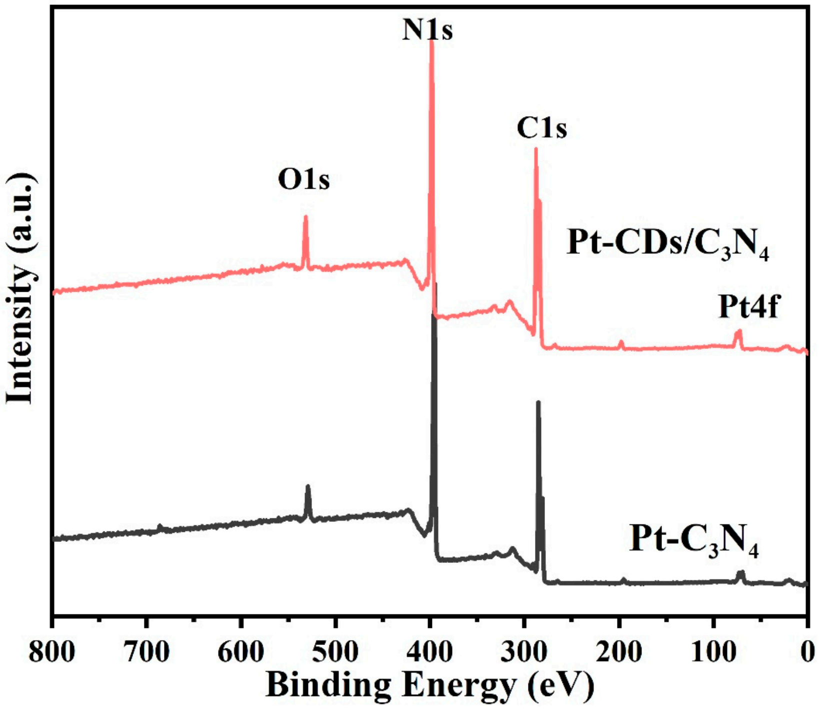

Figure S9. XPS survey spectrum of Pt-C_3N_4 and Pt-CDs/ C_3N_4.

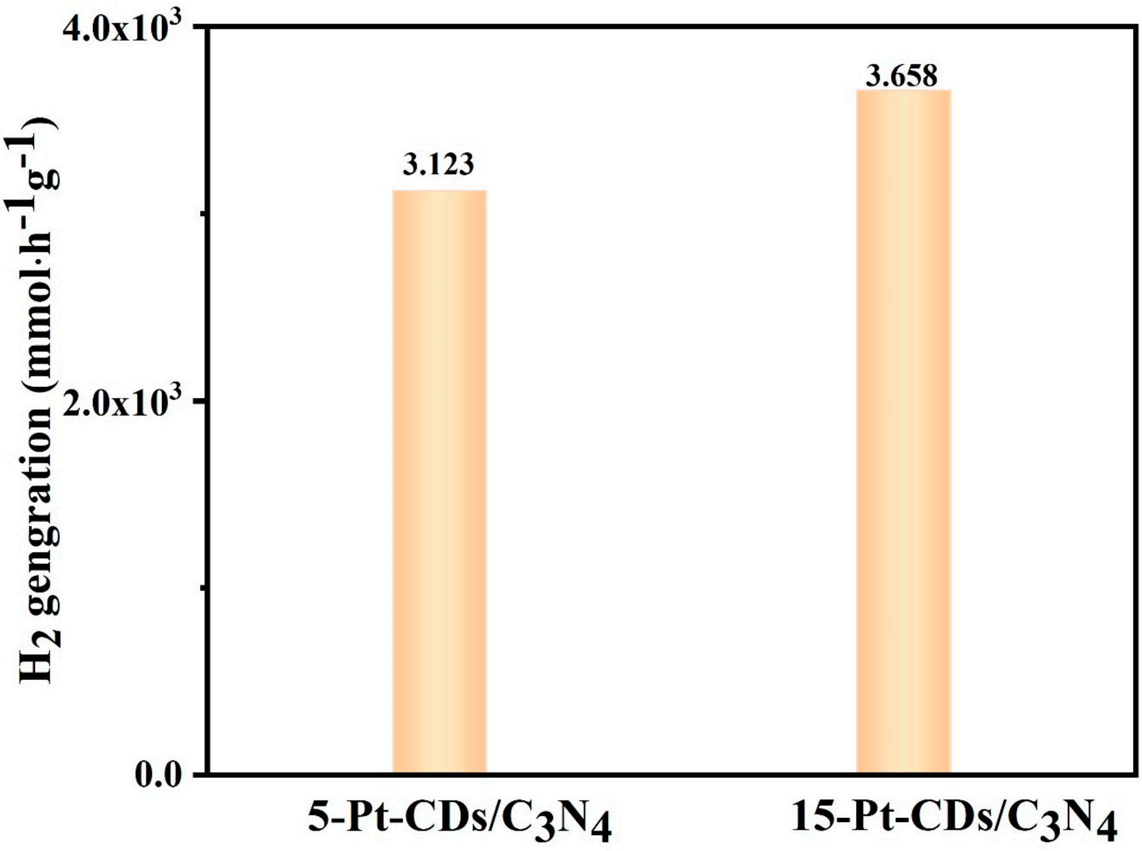

Figure S10. Photocatalytic H_2 evolution rate for 5-Pt-CDs/ C_3N_4 and 15-Pt-CDs/C_3N_4.

Table S1. Type and contents of elements of different samples.

Samples	Type and contents of elements (%)		
		C	
	N-C=N	C-NH$_X$	C-C
C_3N_4	61.7	4.8	33.5
CDs/C_3N_4	48	6.5	45.5

Table S2. Type and contents of elements of different samples.

Samples	Type and contents of elements (%)		
		Nitrogen	
	C-N=C	N-(C)$_3$	C-NH
C_3N_4	72.7	13.0	14.3
CDs/C_3N_4	73.7	10.4	15.9

Table S3. PL lifetime of photogenerated charge carrier

Samples	τ_1 (ns) [A_1]	τ_2 (ns) [A_2]	τ_{Ave} (ns)
C_3N_4	2.9575 [48.56]	12.6506 [51.44]	7.94
CDs/C_3N_4	2.7788 [40.30]	15.3544 [59.7]	10.28

Table S4. EXAFS fitting parameters at the Pt L3-edge for various samples (S_0^2=0.925).

Sample	Shell	CN[a]	R(Å)[b]	σ^2 (Å^2·10^{-3}) [c]	ΔE_0(eV)[d]	R factor (%)
Sample-Pt	Pt-N	4.5±0.19	2.14±0.02	0.003±0.004	7.54±2.53	1.57

Table S5. Photocatalytic hydrogen evolution performance for Pt-CDs/C_3N_4 composite in comparison with other CN-based photocatalysts that reported in recent years.

Photocatalyst	Experimental details (electron donor, co catalyst, light source)	H_2 productivity (mmol $h^{-1}g^{-1}$)	Ref.
CDs/C_3N_4	TEOA, Pt (1 wt%), 300W Xenon lamp, AM1.5	15.09	This work
PCN/NCDs	TEOA, Pt(1 wt%), 300 W Xenon Lamp, λ >420nm	3.731	[22]
HCNS – $C_{1.0}$	TEOA, Pt(3 wt%), 300 W Xenon Lamp, λ >420nm	2.322	[49]
CDs/S-HTCN	TEOA, Pt (1 wt%), 300W Xenonlamp, AM1.5	9.284	[50]
GICN	TEOA, Pt (3 wt%), 300 W Xenon Lamp, λ >420nm	2.684	[13]
P/UH-CNS	TEOA, Pt (2 wt%), 300 W Xenon Lamp, λ > 420nm	2.814	[51]
3DOM V-CN	TEOA, Pt (3 wt%), 300 W Xenon Lamp, λ > 420nm	2.3	[10]
